# Supplementary material for: Identification of a monoclonal antibody that targets PD-1 in a manner requiring PD-1 Asn58 glycosylation
Source: Commun Biol. 2019 Oct 25;2:392. doi: 10.1038/s42003-019-0642-9 (PMC6814707; doi:10.1038/s42003-019-0642-9)
Supplement: Supplementary file 1 — Supplementary information [file 42003_2019_642_MOESM1_ESM.pdf]

Supplementary information

Supplementary Figures

Supplementary Figure 1

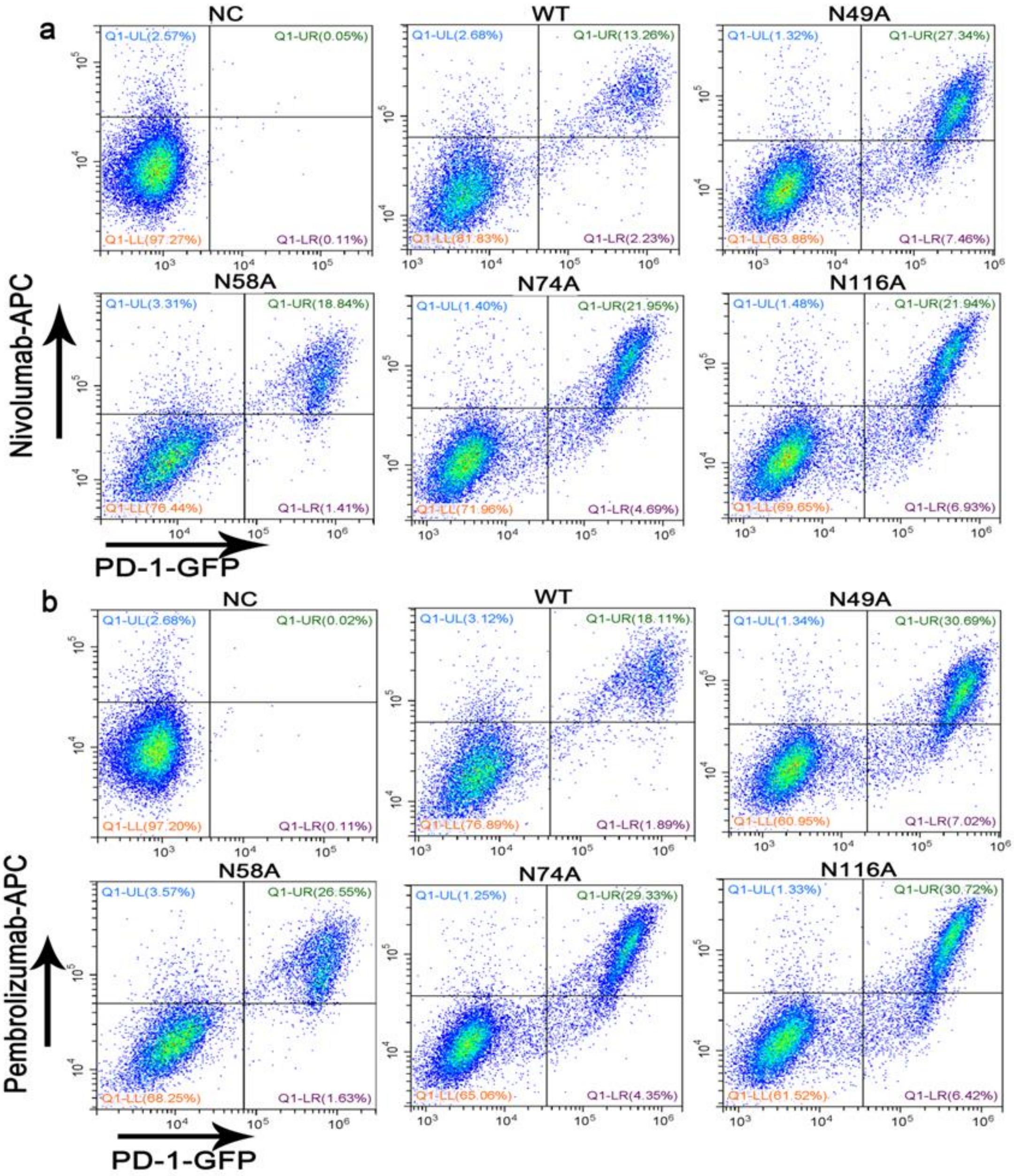

Supplementary Figure 1. N-glycosylation of PD-1 had no effect on the interaction with nivolumab (a) and pembrolizumab (b). A flow cytometric analysis of antibodies binding to WT PD-1 or various glycosylation sites mutated proteins (N49A, N58A, N74A, and N116A) expressed on the cell surface of HEK 293 cells. Plasmids expressing WT PD-1 or mutant proteins fused with EGFP were used for transfection. Mock-transfected HEK 293 cells were used as negative control (NC).

Supplementary Figure 2

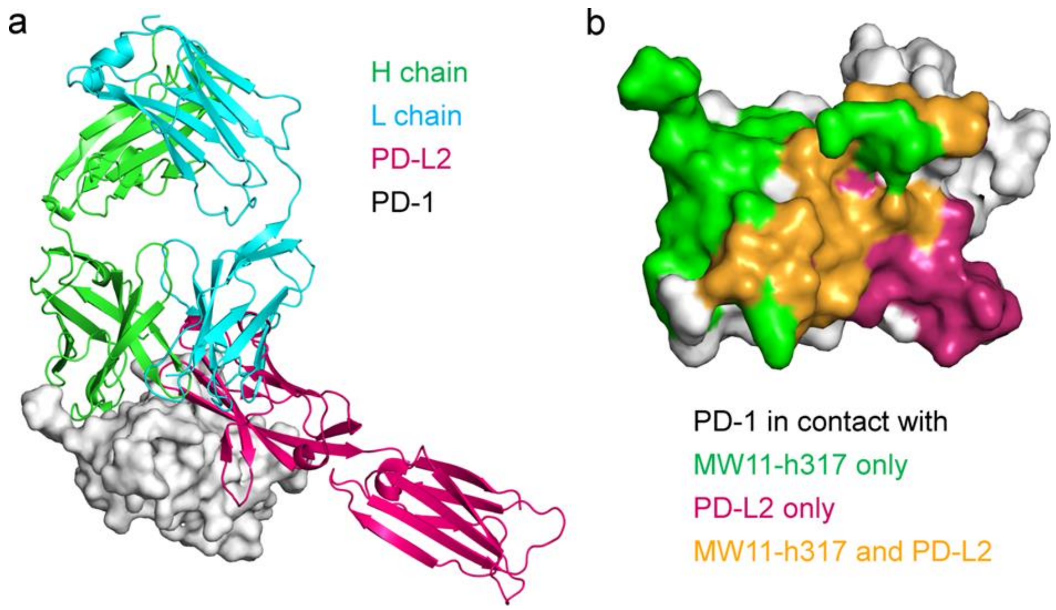

Supplementary Figure 2. Competitive binding of MW11-h317 Fab with PD-1 ligand PD-L2. (a) Superposition of the PD-1/ MW11-h317 Fab complex structure with PD-1/PD-L2 complex structure (PDB ID: 3BP5). MW11-h317 H chain is shown in green, L chain in cyan and PD-L2 in magenta. PD-1 is shown as surface representation in gray. (b) Binding surface of PD-1 with MW11-h317 or PD-L2. The residues in contact with MW11-h317 are shown in green, whereas residues in contact with PD-L2 are shown in magenta, and the overlapping residues bounded by both MW11-h317 and PD-L2 are shown in orange.

Supplementary Tables

Supplementary Table 1 PD-1 binding epitopes in three antibodies.

**Antibody**		**PD-1 binding epitopes**
Pembrolizumab	H chain (VH)	C'D loop; C, C' and F strands
	L chain (VL)	C'D loop
Nivolumab	H chain (VH)	BC loop; N-loop; FG loop
	L chain (VL)	FG loop
MW11-h317	H chain (VH)	BC loop; C'D loop; FG loop; N58 glycosylation
	L chain (VL)	FG loop

Supplementary Table 2 Drug administration design

Group	Number of animals	Drug administration group	Dose (mg/kg)	Administration route	Administration duration (weeks)
1	8	hIgG control	10	i.p.	BIW×3
2	8	MW11-h317-H	10	i.p.	BIW×3
3	8	MW11-h317-M	2	i.p.	BIW×3
4	8	MW11-h317-L	0.5	i.p.	BIW×3
5	8	Nivolumab-L	2	i.p.	BIW×3
6	6–8	Nivolumab-H	10	i.p.	BIW×3

i.p: intraperitoneal injection；BIW: biweekly
