# Supplementary material for: Identification of a monoclonal antibody that targets PD-1 in a manner requiring PD-1 Asn58 glycosylation
Source: Commun Biol. 2019 Oct 25;2:392. doi: 10.1038/s42003-019-0642-9 (PMC6814707; doi:10.1038/s42003-019-0642-9)
Supplement: Supplementary file 2 — Description of additional supplementary items [file 42003_2019_642_MOESM2_ESM.docx]

**Additional Supplementary files**

Supplementary Data 1. Source data for Figure 6e and 7a-d.
