# Supplementary material for: Identification of a monoclonal antibody that targets PD-1 in a manner requiring PD-1 Asn58 glycosylation
Source: Commun Biol. 2019 Oct 25;2:392. doi: 10.1038/s42003-019-0642-9 (PMC6814707; doi:10.1038/s42003-019-0642-9)
Supplement: Supplementary file 4 — Reporting Summary [file 42003_2019_642_MOESM4_ESM.pdf]

nature research

Corresponding author(s): Jinchao Zhang, Min Zhang

Last updated by author(s): Sep 1, 2019

Reporting Summary

Nature Research wishes to improve the reproducibility of the work that we publish. This form provides structure for consistency and transparency in reporting. For further information on Nature Research policies, see Authors & Referees and the Editorial Policy Checklist.

Statistics

For all statistical analyses, confirm that the following items are present in the figure legend, table legend, main text, or Methods section.

n/a	Confirmed	
☐	☒	The exact sample size (*n*) for each experimental group/condition, given as a discrete number and unit of measurement
☐	☒	A statement on whether measurements were taken from distinct samples or whether the same sample was measured repeatedly
☐	☒	The statistical test(s) used AND whether they are one- or two-sided<br>*Only common tests should be described solely by name; describe more complex techniques in the Methods section.*
☐	☒	A description of all covariates tested
☐	☒	A description of any assumptions or corrections, such as tests of normality and adjustment for multiple comparisons
☐	☒	A full description of the statistical parameters including central tendency (e.g. means) or other basic estimates (e.g. regression coefficient) AND variation (e.g. standard deviation) or associated estimates of uncertainty (e.g. confidence intervals)
☒	☐	For null hypothesis testing, the test statistic (e.g. *F*, *t*, *r*) with confidence intervals, effect sizes, degrees of freedom and *P* value noted<br>*Give P values as exact values whenever suitable.*
☒	☐	For Bayesian analysis, information on the choice of priors and Markov chain Monte Carlo settings
☒	☐	For hierarchical and complex designs, identification of the appropriate level for tests and full reporting of outcomes
☒	☐	Estimates of effect sizes (e.g. Cohen's *d*, Pearson's *r*), indicating how they were calculated

Our web collection on statistics for biologists contains articles on many of the points above.

Software and code

Policy information about availability of computer code

Data collection	StudyDirector, version no.: 3.1.399.19; Studylog System, Inc.
Data analysis	HKL2000, Otwinowski and Minor, 1997 http://www.hkl-xray.com/; PHASER, McCoy et al., 2007, http://www.ccp4.ac.uk/; COOT, Emsley and Cowtan, 2004, http://www2.mrc-lmb.cam.ac.uk/Personal/pemsley/coot/; REFMAC, Murshudov et al., 2011 http://www.ccp4.ac.uk/; PHENIX, Adams et al., 2010 http://www.phenix-online.org/

For manuscripts utilizing custom algorithms or software that are central to the research but not yet described in published literature, software must be made available to editors/reviewers. We strongly encourage code deposition in a community repository (e.g. GitHub). See the Nature Research guidelines for submitting code & software for further information.

Data

Policy information about availability of data

All manuscripts must include a data availability statement. This statement should provide the following information, where applicable:

- Accession codes, unique identifiers, or web links for publicly available datasets
- A list of figures that have associated raw data
- A description of any restrictions on data availability

PDB 6JJP

Field-specific reporting

Please select the one below that is the best fit for your research. If you are not sure, read the appropriate sections before making your selection.

☒ Life sciences ☐ Behavioural & social sciences ☐ Ecological, evolutionary & environmental sciences

For a reference copy of the document with all sections, see nature.com/documents/nr-reporting-summary-flat.pdf

Life sciences study design

All studies must disclose on these points even when the disclosure is negative.

Sample size	In vivo anti tumor experiment, eight mice used in each group
Data exclusions	No data were excluded from analyses.
Replication	All attempts at replication were successful.
Randomization	All mice were randomly divided into six groups for drug administration.
Blinding	The investigators were blinded to group allocation during data collection and analysis.

Reporting for specific materials, systems and methods

We require information from authors about some types of materials, experimental systems and methods used in many studies. Here, indicate whether each material, system or method listed is relevant to your study. If you are not sure if a list item applies to your research, read the appropriate section before selecting a response.

Materials & experimental systems

n/a	Involved in the study	
☐	☒	Antibodies
☐	☒	Eukaryotic cell lines
☒	☐	Palaeontology
☐	☒	Animals and other organisms
☒	☐	Human research participants
☒	☐	Clinical data

Methods

n/a	Involved in the study	
☒	☐	ChIP-seq
☐	☒	Flow cytometry
☒	☐	MRI-based neuroimaging

Antibodies

Antibodies used	Alexa Fluor® 647 anti-human IgG: Jackson Immuno Research, Cat# 109-605-098;<br>HRP -anti-mouse Fc secondary antibodies: Jackson Immuno Research, Cat# 115-035-071;<br>human NC-IgG4: Shanghai Destiny Biotech Co., Ltd, Lot: AB170090;<br>nivolumab: Bristol-Myers Squibb, Lot: AAW4553;<br>pembrolizumab : Merck &Co., Lot: 6SNL81506<br>MW11-h317: This study
Validation	MW11-h317 and Nivolumab both are humanized antibodies

Eukaryotic cell lines

Policy information about cell lines

Cell line source(s)	CHO-K1: ATCC, CCL-61; FreeStyle 293-F: Invitrogen, R79007;
Authentication	The cell lines were purchased and authenticated from ATCC or Invitrogen.
Mycoplasma contamination	All cell lines were negative for mycoplasma contamination
Commonly misidentified lines (See ICLAC register)	No commonly misidentified cell lines used

Animals and other organisms

Policy information about studies involving animals; ARRIVE guidelines recommended for reporting animal research

Laboratory animals	All mice used for the present study were C57BL/6J mice of female (6-8 weeks old).
Wild animals	The study did not involve wild animals.
Field-collected samples	The study did not involve samples collected from the field.
Ethics oversight	Animal experiments were approved by Institutional Animal Care and Use Committee (IACUC) of Crown Bioscience (Taicang) Inc. The approval number is AN-1803-13-502.

Note that full information on the approval of the study protocol must also be provided in the manuscript.

Flow Cytometry

Plots

Confirm that:

☒ The axis labels state the marker and fluorochrome used (e.g. CD4-FITC).

☒ The axis scales are clearly visible. Include numbers along axes only for bottom left plot of group (a 'group' is an analysis of identical markers).

☒ All plots are contour plots with outliers or pseudocolor plots.

☒ A numerical value for number of cells or percentage (with statistics) is provided.

Methodology

Sample preparation	MW11-h317 used was prepared by Jiangsu T-mab BioPharma Co., Ltd, subcompany of Mabwell (Shanghai) Bioscience Co., Ltd. recombinant PD-1 and h317-Fab were prepared by Beijing Kohnoor Science &Technology Co., Ltd
Instrument	Octet QKe system (Fortebio) used for affinity detection;<br>BECKMAN COULTER/ B49009AD (CytoFLEX FACS machine) used for FACS;<br>HPLC Arc(2489 K16VTU290A ; Arc FTN M15VSM294N ;Arc QSM K16VQS253G,water) used for antigen-antibody complex analysis;<br>Octet QKe system (Fortebio) used for affinity detection;<br>BECKMAN COULTER/ B49009AD (CytoFLEX FACS machine) used for FACS;<br>HPLC Arc(2489 K16VTU290A ; Arc FTN M15VSM294N ;Arc QSM K16VQS253G,water) used for antigen-antibody complex analysis;
Software	Date Analysis 9.0 (Fortebio) used to calculate the kinetic constant of the antigen-antibody binding;<br>CytExpert 2.3 for FACS analysis;<br>Empower3 for HPLC;
Cell population abundance	the PD-1-GFP positive cells population used for analyzing the binding of mAbs with WT or mutant PD-1. So the PD1-GFP negative cells population abundance is not necessary for analyzing.
Gating strategy	Main cells mass gated out for mapping the result figures. In representative figure here, cell population in Q1LL was PD1-GFP negative cells while cell population in Q1UR or Q1LR was PD1-GFP positive cells.

☐ Tick this box to confirm that a figure exemplifying the gating strategy is provided in the Supplementary Information.
